# A Manual Transportable Instrument Platform for Ground-Based Spectro-Directional Observations (ManTIS) and the Resultant Hyperspectral Field Goniometer System

**DOI:** 10.3390/s131216105

**Published:** 2013-11-26

**Authors:** Marcel Buchhorn, Reinhold Petereit, Birgit Heim

**Affiliations:** 1 Alfred Wegener Institute Helmholtz Centre for Polar and Marine Research (AWI), Research Unit Potsdam, Telegrafenberg A43, Potsdam D-14473, Germany; E-Mail: birgit.heim@awi.de; 2 Alfred Wegener Institute Helmholtz Centre for Polar and Marine Research (AWI), Scientific Workshop Department Bremerhaven, Am Handelshafen 12, Bremerhaven D-27570, Germany; E-Mail: reinhold.petereit@awi.de

**Keywords:** reflectance anisotropy, spectro-directional remote sensing, spectro-goniometer, HDRF, BRDF, hyperspectral HCRF measurements

## Abstract

This article presents and technically describes a new field spectro-goniometer system for the ground-based characterization of the surface reflectance anisotropy under natural illumination conditions developed at the Alfred Wegener Institute (AWI). The spectro-goniometer consists of a *Man*ual *T*ransportable *I*nstrument platform for ground-based *S*pectro-directional observations (ManTIS), and a hyperspectral sensor system. The presented measurement strategy shows that the AWI ManTIS field spectro-goniometer can deliver high quality hemispherical conical reflectance factor (HCRF) measurements with a pointing accuracy of ±6 cm within the constant observation center. The sampling of a ManTIS hemisphere (up to 30° viewing zenith, 360° viewing azimuth) needs approx. 18 min. The developed data processing chain in combination with the software used for the semi-automatic control provides a reliable method to reduce temporal effects during the measurements. The presented visualization and analysis approaches of the HCRF data of an Arctic low growing vegetation showcase prove the high quality of spectro-goniometer measurements. The patented low-cost and lightweight ManTIS instrument platform can be customized for various research needs and is available for purchase.

## 1. Introduction

Spectro-directional remote sensing (RS) has become more and more important in recent years [1,2]. The angular information source can be used to minimize the impact of reflectance anisotropy in RS data of sensor systems with pointing capabilities or wide swaths achieving high quality, consistent and therefore comparable and reproducible data sets [2]. Moreover, various studies have shown that canopy architecture properties can be derived from spectro-directional RS data [3–8]. The directional reflectance properties of a surface are mathematically specified by the bidirectional reflectance distribution function (BRDF) [9]. Direct BRDF measurements are not possible, but the anisotropic reflectance behavior of a surface can be approximately determined by measuring the hemispherical conical reflectance factor (HCRF) in the field. Therefore, various types of ground-based measurement instrumentation called field spectro-goniometers have been developed in the recent years [10–21].

Field spectro-goniometers are used as a tool to provide spectro-directional characteristics of various surfaces for: (i) the investigation of the physical mechanism of BRDF effects; (ii) the development and validation of BRDF models; (iii) the investigation of the relationship between BRDF effects and biophysical parameters; as well as (iv) the validation of satellite and aircraft based BRDF data [22]. Up to now, most of the developed field spectro-goniometer systems, due to their design, are not applicable in geographical and logistical challenging regions such as the Arctic or on permafrost surfaces. Performing spectro-goniometer measurements in these challenging regions demands specific technical requirements such as: (i) a lightweight construction; (ii) a low-cost production; (iii) standard parts for easy replacement; (iv) a disassembly and storage in boxes for transport by small helicopters with a helicopter sling or on sleds; and (v) a secure footing on small building areas. But at the same time the design and sensor configuration has to be robust enough to allow observations with: (i) a high angular accuracy; (ii) a minimum distance of 2 m between the vegetated surface and the sensor; (iii) a constant observation center; (iv) a fast scanning in all directions reducing the impact of temporal illumination changes; and (v) a high spectral resolution [22,23]. Moreover, a high level of automation of the measurement process used by field spectro-goniometers such as the dual-view FIGOS [15], the IAC ETH goniospectrometer [16], the PARABOLA III [13], the ASG [18], or the FIGIFIGO [17] is expensive with respect to the development of the sensor and control systems, and also may be susceptible to damage in geographical regions with fast changing weather conditions.

Following these requirements, a spectro-goniometer system was developed at the Alfred Wegener Institute (AWI) for the spectro-directional characterization of low-growing vegetation communities in the Arctic. This system consists of a low-cost, lightweight instrument platform for the angular positioning of the sensor within 30° view zenith and 360° view azimuth angle, and a hyperspectral sensor system including two spectro-radiometers for the radiance and irradiance measurements. The sensor system itself can be customized to the research needs. The reason for the smaller defined view zenith pointing capability is that many present and upcoming satellite sensors such as RapidEye [24], Environmental Mapping and Analysis Program (EnMAP) [25], and PRecursore IperSpettrale of the application mission (PRISMA) [26] have a maximal off-nadir tilting of ±30° (RapidEye ±25°, EnMAP ±30°, PRISMA ±15°). Moreover, this pointing capability is adequate for BRDF analysis in multi-angle data sets created by successive passes of satellite sensor systems with nadir pointing, or for the BRDF normalization in RS data acquired by satellite sensors with wide swaths.

The emphasis of this article is the presentation and technical description of the *Man*ual *T*ransportable *I*nstrument platform for ground-based *S*pectro-directional observations (called ManTIS) as well as the description of the sensor system used for the resultant hyperspectral field spectro-goniometer system. Moreover, we present the measurement strategy for HCRF acquisitions in the field in connection with an error assessment as well as the processing and visualization of the HCRF data. Finally, the HCRF measurements of an example surface are processed, presented and discussed.

## 2. Theoretical Background

**Figure 1.**
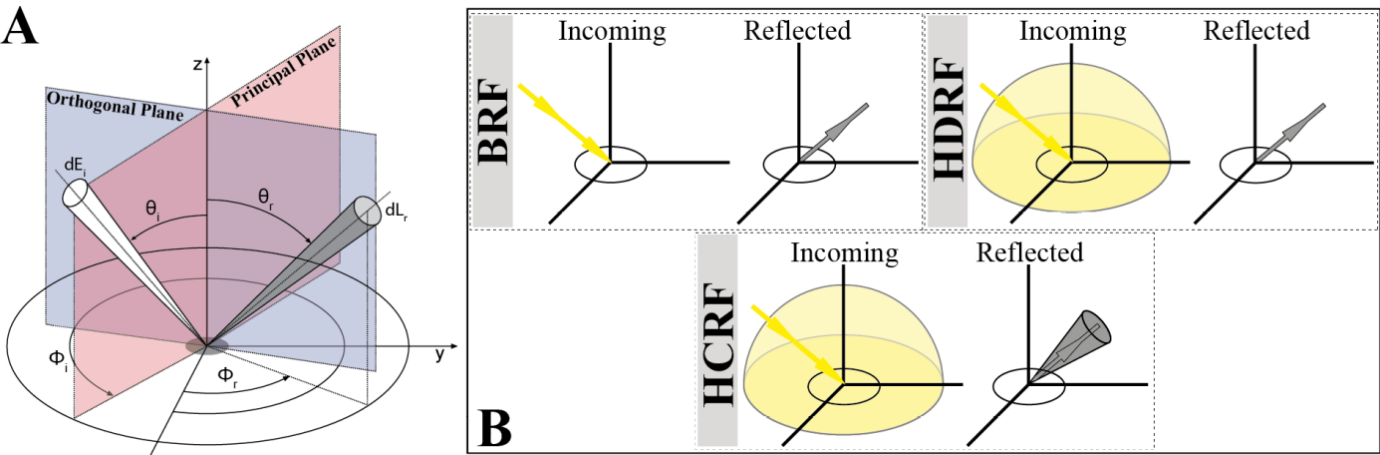
(**A**) Concept of the bidirectional reflectance distribution function (BRDF) [9]; (**B**) Reflectance nomenclature as a function of geometrical aspects used in this study [33].

Natural surfaces do not show Lambertian reflectance behavior [7,27–32], and instead they display anisotropic reflectance distributions which affect all remotely sensed radiation data. This reflectance anisotropy is based on the properties of the observed surface and can be physically described by a set of functions (ƒ_r_ [sr^−1^]) characterizing the reflected radiation as a function of the incident beam [33]. In detail, according to Nicodemus *et al.* [9] this so called BRDF is defined as the ratio of the radiance dL_r_ (W·m^−2^·nm^−1^·sr^−1^) reflected from the surface in one direction (θ_r_, ϕ_r_) to the incident irradiance dE_i_ (W·m^−2^·nm^−1^) illuminating the surface outgoing from direction (θ_i_, ϕ_i_). Since diffuse reflection causes the incident radiance dL_i_ to be reflected in all directions over the hemisphere, the BRDF uses the incident irradiance dE_i_ and is not dimensionless, and therefore measured in sr^−1^. This relationship is visualized in Figure 1A and mathematically expressed in Equation (1):

(1) $${\text{BRDF}}_{\lambda }=f_{r}(\lambda ;\theta_{i},\phi_{i};\theta_{r},\phi_{r})=\frac{dL_{r}(\lambda ;\theta_{i},\phi_{i};\theta_{r},\phi_{r})}{dE_{i}(\lambda ;\theta_{i},\phi_{i})}\left[sr^{-1}\right]$$

Furthermore, the BRDF is not only dependent on the illumination and viewing directions each identified by two angles, the illumination (resp. viewing) zenith angle θ_i_ (resp. θ_r_) and the illumination (resp. viewing) azimuth angle ϕ_i_ (resp. ϕ_r_), but also depends on the wavelength (λ) and polarization of the electromagnetic radiation.

For practical reasons the bidirectional reflectance factor (BRF) is used to describe the reflectance anisotropy of a surface. The BRF can be estimated by the ratio of the radiance L_r_ reflected from the surface in a specific direction to the radiance L_ref_ reflected from a lossless reference panel with Lambertian reflectance behavior, both measured under identical illumination geometry [9]. Since white reference panels like Spectralon^®^ panels do not show ideal Lambertian reflectance characteristics, the radiance L_ref_ has to be corrected by a panel calibration coefficient R_ref_. BRF measurements like those shown in Equation (2) are dimensionless and interrelated to the BRDF. Under the assumption that the irradiance is isotropic and that the BRDF is constant within the illumination-sensor-geometry, the BRF divided by π approximately reproduces the BRDF of the surface [33]:

(2) $$\text{BRF}(\lambda ;\theta_{i},\phi_{i};\theta_{r},\phi_{r})=\frac{L_{r}(\lambda ;\theta_{i},\phi_{i};\theta_{r},\phi_{r})}{L_{\text{ref}}(\lambda ;\theta_{i},\phi_{i};\theta_{r},\phi_{r})}.R_{\text{ref}}(\lambda ;\theta_{i},\phi_{i};\theta_{r},\phi_{r})$$

For BRF measurements under field conditions, Equation (2) is still not applicable. Since the illumination is hemispherical under natural illumination conditions, the best estimation for the BRF would be the measurement of the hemispherical directional reflectance factor (HDRF) [9,33]. Exact HDRF measurements would require a sensor optic with infinitesimally small instantaneous field of view (IFOV) which is impossible to obtain. The best estimation of reflectance anisotropy in the field is therefore the measurement of the HCRF [9,33]. Figure 1B shows the relation of incoming and reflected radiance terminology used to describe the three (BRF, HDRF, and HCRF) reflectance quantities [33].

Since the spectro-radiometers considered for the ManTIS field spectro-goniometer uses foreoptics with an IFOV smaller than 10° [21], the observation geometry of the sensor is conical. Under the assumption that the HCRF is constant over the IFOV of the sensor, we could equate our HCRF measurements with the HDRF [1]. Different publications have shown that this is done for sensor IFOVs smaller than 3° [14–16], but in our case it has still to be proven. Therefore, to avoid misunderstandings, we want to clarify that the spectro-directional measurements with the ManTIS field spectro-goniometer in its current sensor configuration are HCRF measurements.

## 3. Description of the Field Spectro-Goniometer System

### 3.1. Construction Schedule

The preliminary considerations for a field spectro-goniometer platform started in October 2010 and were followed by a two month design-related period. The main focus during this design phase was on the transportability and lightweight construction of the prototype which was built from January to February 2011 by the scientific workshop of the AWI in Bremerhaven, Germany. Afterwards, the prototype ran through the first field experiments. These initial tests showed promising results, but also exposed room for improvements. In March 2011, we started with the revision of the structural design of the platform. Therefore, a computer-based 3D model was created and optimized with the help of a ray tracing simulation. The construction phase of the revised version of the prototype ran from May to June 2011. Afterwards the ManTIS was equipped with a customized sensor system, calibrated and tested at the AWI facility in Potsdam, Germany. In July 2011, the AWI ManTIS field spectro-goniometer became fully operational and was packed for its first Arctic mission on the Yamal 2011 expedition (25 July to 9 September 2011) to the Yamal Peninsula, West-Siberia, Russia [34].

During the Yamal expedition, the field spectro-goniometer was extensively tested under Arctic conditions and showed excellent results. Nevertheless, the selected design showed also some challenges in the assembly of the system prior to the measurements. The Arctic conditions demand an easy assembly without tools for screws and nuts as well as require adjustment wheels on setting screws which can be handled with gloves. Therefore, we decided to revise the design of the ManTIS prototype once more and additionally bring the prototype to maturity phase. All parts were built now by computerized numerical control (CNC) machines. This improvement allows a fast reproducibility of missing parts or the whole instrument platform (mass production). Moreover, plug-and-socket connections with self-locking screws and nuts allow now a faster assembly in the field and an improved stability under load on permafrost surfaces. The design phase of the improved ManTIS ran from October to December 2011, where the main construction phase ran from January to April 2012. Again, all parts were built and pre-assembled by the scientific workshop of the AWI in Bremerhaven, Germany. In May 2012, the field spectro-goniometer system was calibrated and tested for the next field season at the AWI facility in Potsdam, Germany. By the end of May 2012 the improved ManTIS field spectro-goniometer reached its final stage of extension and became fully operational. Once more, it was packed for detailed field tests on the Alaska 2012 expedition (21 June to 22 July 2012) to the North Slope, AK, USA [35]. Overall, the design, construction and setup of the ManTIS field spectro-goniometer has required about 18 months with approx. 1,500 working hours and additional 135 machine hours.

### 3.2. Description of the Field Spectro-Goniometer Platform (ManTIS)

**Figure 2.**
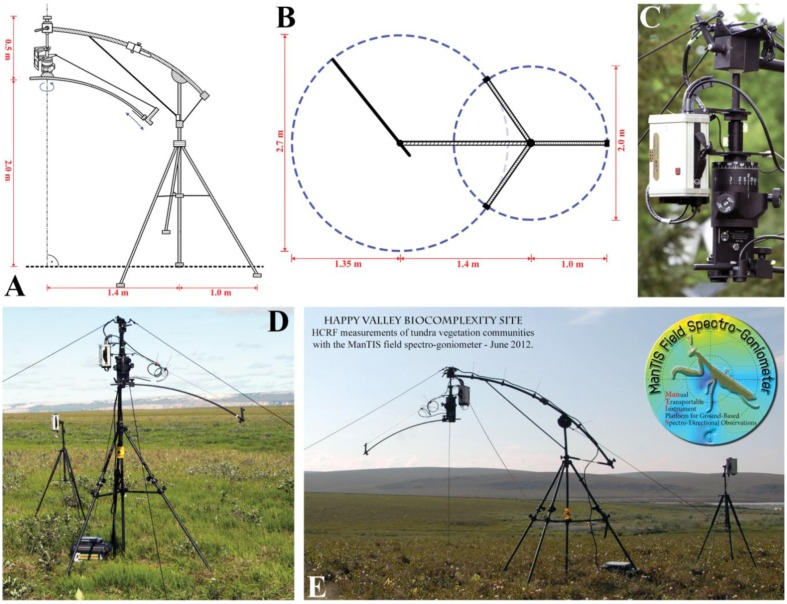
(**A**) Design and dimensions of the ManTIS (front view); (**B**) Design and dimensions of the ManTIS (top view); (**C**) The suspension including the azimuth angle adjustment module (AAM) with connected GER-1500 spectro-radiometer; (**D**) ManTIS field spectro-goniometer (lateral view); (**E**) Overview of ManTIS field spectro-goniometer assembled for a field campaign in the Alaskan Low Arctic showing both GER-1500 spectro-radiometers (front view).

The field spectro-goniometer platform consists of five major parts: a tripod with a stabilized center post; a cantilever connected to the center post and stabilized by bracings; a rotatable and fixable suspension including the azimuth angle adjustment module (AAM) connected to the cantilever; a zenith arc with one end connected to the suspension; and a sensor sled which slides on the zenith arc (Figure 2A). All components are made of black anodized aluminum, reducing the overall weight to only 27 kg (without the sensor system). The complete ManTIS can be disassembled and stored in a box with the dimensions of 146 × 47 × 29 cm, increasing the overall weight then to 42 kg. This weight and box size permits the transport in station wagons and as normal luggage in passenger planes and trains, and therefore allows for fast and convenient access to logistically demanding study sites.

The tripod design was chosen because it keeps the center post in its vertical position and provides the best weight-to-stability ratio against downward and horizontal forces. Moreover, it can be setup and leveled in all kinds of Arctic environments. The feet of the tripod are formed as floor plates. Depending on the substrate, special shoes can be attached on the floor plates to improve stability, e.g., for permafrost surfaces the shoes have a drift pin. The cantilever consists of two slightly bent tubes which can be folded up, and is fixed on the upper end of the center post. Moreover, the cantilever is laterally braced to the center post. Through adjustments of the bracings of the cantilever, the overall distance between the surface and the zenith arc can be set. The suspension is connected via a ball-and-socket join to the other end of the cantilever. This connection allows the exact leveling of the suspension in the vertical center line of the target (correspond to the nadir view position), and therefore also permits spectro-goniometer measurements in rough terrain. The other end of the suspension contains the AAM which has a fixed square joint to the end of the zenith arc (Figure 2C). To stabilize this connection, the other end of the zenith arc is connected to the middle part of the suspension via a bracing, which further helps to guide electrical and optical cables to the sensor sled. Important to mention is that the center post, the cantilever and the suspension are matched to each other so that the zenith arc is positioned at the distance of the arc radius (2.05 m) above the ground. All mounting and adjustment screws are made of steel but with large adjustment wheels made of cold-resistant plastic.

Since the zenith arc can be fully rotated about the center line of the AAM and the sensor sled can be positioned on the zenith arc in any off-view angle up to 30°, the spectro-radiometer connected to the sensor sled of the ManTIS can measure the target with view zenith angles from −30° to +30° and at all desired view azimuth angles. Moreover, this setting allows measurements with a constant observation center. The angular positioning of the sled is carried out manually; consequently the zenith arc has engraved labels with a resolution of 1°. To decrease the time for repositioning the view azimuth angle, the AAM has an internal quick-action locking system. In detail, preferred view azimuth angles (up to two different measurement schemes) can be engraved on a cylinder in the inner core of the AAM. Through a locking screw, the zenith arc can be fixed in an azimuth plane, preferably the solar principal plane. Outgoing from this azimuth plane, the zenith arc can then only be rotated in the azimuthally angular distances provided by the engraved measurement scheme. The measurement scheme itself can be chosen by two additional set screws. A 360° scale engraved on an additional outer ring of the AAM also allows the view azimuth angles to be freely set with respect to the solar principal plane. Also important to mention is that in order to measure in the solar principal plane, the zenith arc has been mounted eccentrically on the AAM and only the sensor sled itself moves directly in the solar principal plane. In order to help to set the zenith arc into the solar principal plane, the AAM has an additional second outer ring with a 360° engraved scale which can be orientated to geographic north by a compass. This second outer ring is independent and does not rotate when the zenith arc is moved.

### 3.3. Sensor Configuration of the AWI ManTIS Field Spectro-Goniometer

The ManTIS was designed as a platform which can be equipped with various kinds of spectro-radiometers and other hardware in order to form field spectro-goniometer systems customized to the specific research needs. In its current configuration the platform was modified for EnMAP purposes and is equipped with two PC-controlled GER-1500 portable spectro-radiometers (Spectra Vista Corporation, Poughkeepsie, NY, USA), a Navilock NL-402U global positioning system (GPS) receiver (Tragant GmbH, Berlin, Germany), a NC-Eye camera system designed for Arctic environments (AnKoTec Anton Kothe, Postbauer-Heng, Germany), and a 5 × 5 inches Spectralon® white panel with >99% diffuse reflectance standard (Labsphere, Inc., North Sutton, NH, USA).

The GER1500 spectro-radiometers measure radiance across the wavelength range of 350–1,050 nm with sampling intervals of 1.5 nm [36], and are connected via serial cables (nine-pin RS-232) to a field computer. One GER-1500 is mounted to the suspension of the ManTIS and measures the radiance reflected from the target surface (Figure 2C–E). The foreoptic of this spectro-radiometer is mounted to the sensor sled and connected via a 1.6 m long fiber optic cable. In its current configuration, the foreoptic has an IFOV of 8.5°. In order to measure the radiance reflected from a Spectralon® reference panel, a sub-arm with a mounting clip can be attached to the sensor sled allowing precise alignment of the reference panel to the vertical center line of the target surface in the nadir measurement position.

Additionally, a video camera connected via universal serial bus (USB) cable to the field computer is mounted on the sensor sled next to the foreoptic of the spectro-radiometer. The center of projection of the camera lens can be made to coincide with the center of the ground instantaneous field of view (GIFOV) of the foreoptic via adjustable mounting clamps. The USB GPS receiver is mounted on top of the cantilever in line with the center line of the suspension, providing the exact geographical position of the target surface. The second GER-1500 spectro-radiometer is equipped with a cosine diffusor foreoptic and mounted at a height of 1.80 m on a tripod for measuring the down-welling total irradiance (Figure 2D,E). This extra tripod is placed near the center post of the ManTIS.

All required sensor cables are combined in a cable loom which is guided from the suspension of the ManTIS over the cantilever to the center post. This reduces the risk of cable jams and facilitates quick setup during the assembling stage. Overall, the sensor system including the cable loom has a weight of approx. 7 kg and it stored in a box with the dimensions of 53 × 44 × 22 cm. Therefore, the weight of the AWI ManTIS field spectro-goniometer in its current field configuration (platform + sensor system) is approx. 34 kg. Together with the two transport boxes, the total shipping weight is approx. 54 kg.

The overall dimensions of the field spectro-goniometer can be seen in Figure 2A,B. The maximum height is 2.5 m, where the zenith arc is positioned at a height of 2.05 m and the sensor of the mounted spectro-radiometer is positioned at a constant distance of approx. 2 m from the target. Since the zenith arc rotates around the vertical center line of the target, a sphere of 1.35 m in radius around the target is created. Additional space around the center post (approx. 1 m in radius) is required for the assembly of the tripod. About 45 min are needed for a team of two people to assemble the ManTIS and set up the sensor system. In locations under wind influence, an additional wind brace made of distortion-free rope can be used to further stabilize the field spectro-goniometer (Figure 2D,E).

### 3.4. Measurement Strategy

Due to the relatively small IFOV and the short distance between the foreoptic and the target, the sampling area of the ManTIS field spectro-goniometer is small. In order to acquire representative measurements, targets should be homogeneous surfaces. On the other hand, this small sampling size has the advantage that already homogeneous plots with a size of 1 m × 1 m can be investigated.

In preparation of the spectro-goniometer measurements, the selected sampling plot is marked with small flags in the corners. Next, the center post is positioned at a distance of 1.40 m to the north of the center of the sampling plot and vertically fixed. This prevents a shadowing of the plot by the ManTIS itself. After mounting the cantilever to the center post, the suspension is connected to the cantilever and the zenith arc is locked to the AAM of the suspension. Then the sensor sled is clipped on the zenith arc, and all sensors are mounted. Afterwards, the cable loom is installed and the sensor system is connected to the field computer. Since the center post can be rotated, the assembling can be done outside the sampling plot and the cantilever is then turned towards the target and fixed. This avoids disturbance of the plot during the assembling phase. In order to bring the center line of the AAM in conformity with the vertical center line of the target (nadir view position), final adjustments have to be done at the ball-and-socket join of the suspension with the cantilever. In conclusion, the center of the foreoptic of the spectro-radiometer is now exactly vertical positioned above the center of the sampling plot. By rotating the zenith arc and displacing the sensor sled along the zenith arc, it is possible to position the foreoptic at any point on the spanned spherical shell.

**Figure 3.**
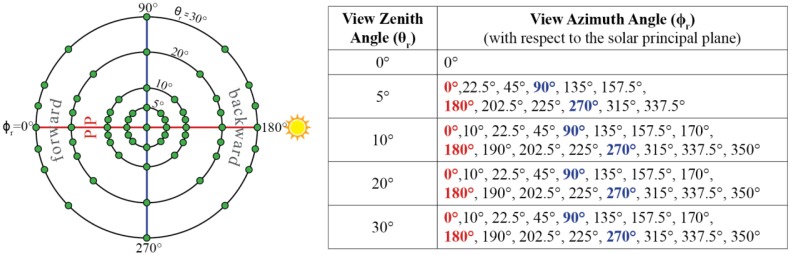
Default measurement scheme of the ManTIS field spectro-goniometer with overall 61 target measurements positions on the spanned spherical shell. The measurement scheme shows a higher measuring density around the solar principal plane (PP).

The design of the measurement scheme as well as the documentation of the sampling plot and measurements follow the recommendation of Sandmeier [22]. In its current configuration, the ManTIS field spectro-goniometer uses a measurement scheme with 61 viewing positions on the spanned spherical shell (Figure 3). Since reflectance anisotropy is more strongly pronounced in the solar principal plane [14,22], the measurement scheme has a higher measuring density around the solar principal plane [21]. In the beginning of each measurement scheme, the zenith arc is aligned with the solar principal plane with the help of the AAM. The first target measurement is taken in the nadir view position. Then the sensor sled is positioned at the 5° view zenith angle position on the zenith arc and target measurements with increasing view azimuth angles are carried out by rotating the zenith arc around the AAM. Afterwards, the sensor sled is positioned to the next view zenith angle position on the zenith arc and the procedure to take target measurements is repeated. Where the target measurements with a 5° view zenith angle are taken at 12 view azimuth angle positions, target measurements with a 10°, 20°, and 30° view zenith angle are taken at 16 view azimuth angle positions.

At the beginning and end of each measurement scheme, the radiance reflected from the Spectralon® reference panel is measured in the nadir view position. Moreover, simultaneously to all target measurements the irradiance profiles are recorded by the second spectro-radiometer with the attached cosine diffusor foreoptic. A video showing the whole measuring process is available on the internet at http://tinyurl.com/ManTISmovie (DOI: 10.1594/PANGAEA.819494). The total acquisition time for this measurements scheme (61 target, two reference panel, and 63 irradiance) is approx. 18 min.

### 3.5. Software for Semi-Automatic Control

**Figure 4.**
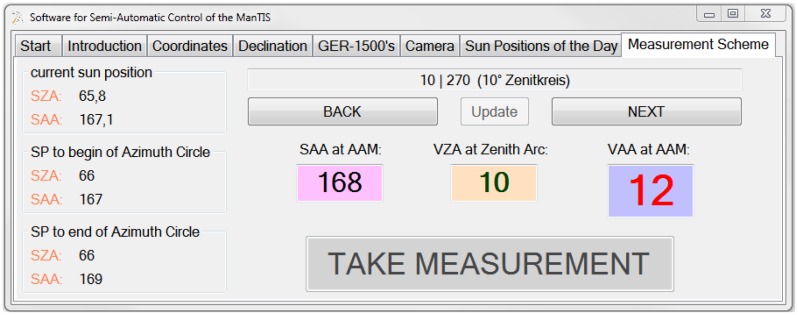
Graphical user interface (GUI) of the software application for the semi-automatic control of the ManTIS field spectro-goniometer.

A software application for the semi-automatic control of the ManTIS field spectro-goniometer was written and coded in visual basic (VB). The graphical user interface (GUI) helps to enter and set the required advance information prior to the measurements. Moreover, the software application calculates from the received GPS information the solar zenith and azimuth position prior to each target measurement. Then the GUI visualizes all configurations for the AAM in order to setup the view zenith and azimuth angles for the target measurements in the selected measurement scheme (Figure 4).

Furthermore, the software communicates with the spectro-radiometers and secures that the radiance and irradiance measurements are taken simultaneously as well as that the received data is correctly named and stored. Additionally, the software controls the video camera system to take a photo of the sampling plot simultaneously along with each target measurement and stores it together with all other data in the database. The generated log file includes all realized software operations with a timestamp.

## 4. Error Assessment

The errors in HCRF acquisitions with the ManTIS field spectro-goniometer can be divided into two broad categories: internal and external error sources. Internal error sources are here defined as measuring inaccuracies through problems with the platform or spectro-radiometer including radiometrical accuracy, white reference calibration, angular accuracy from both positioning and opening angle of the optics, and sensor shadowing. External errors include the variation of incident light through the measurement scheme, environmental influences, representativeness of the sample, and diurnal changes of vegetated surfaces.

### 4.1. Radiometrical Accuracy

The radiometrical accuracy of the spectro-goniometer measurements follows the same principles as any spectro-radiometer measurements, and depends on a good calibration of the devices. In its current configuration, we use two GER-1500 spectro-radiometers which have an average radiance accuracy of 1.2 × 10^−10^ W·cm^−2^·nm^−1^·sr^−1^ [36] (last calibrated in May 2011). By transferring the wavelength dependent radiance accuracy stated in the calibration certificate to the spectro-goniometer measurements in the Arctic, the GER-1500 shows in its wavelength range from 350–1,050 nm a reflectance uncertainty of 0.59% at 400 nm, of 0.20% at 700 nm, and of 1.59% at 900 nm. In order to increase the signal-to-noise ratio, 32 individual measurements are averaged per one target scan.

Since all HCRF values are calculated as a ratio between the radiance reflected of the surface and a Spectralon® white reference panel, errors due to the condition of the panel or a tilt of the reference panel can cause a systematical error [37]. Therefore, regular calibration of the Spectralon® panel at the factory is recommended. In order to decrease tilt errors, we use bubble levels to balance the reference panel. Moreover, Sandmeier *et al.* [38] showed that calibrated Spectralon® panels can even in the nadir view position have changes in the measured radiance depending on the illumination zenith angle. Under the assumption that the reflectance anisotropy is nearly invariant between different Spectralon® panels [39], we use the correction algorithm developed by Sandmeier *et al.* [38] in order to reduce systematical errors. This approach is already tested and used with other spectro-goniometers [14,16].

### 4.2. Pointing Accuracy

The angular accuracy of the spectro-goniometer measurements is defined by the roundness of the zenith arc, the precision in the positioning of the sensor sled on the zenith arc, and the correctness in setting the view zenith angle in the AAM. In case of the ManTIS field spectro-goniometer, an additional factor has to be considered. Since the zenith arc is freely suspended in the center of the sampling plot, care has to be taken that the center line of the AAM (on which the zenith arc is orthogonally mounted) is in conformity with the vertical center line of the target, and also that the zenith arc is positioned at the distance of the arc radius above the ground. Therefore, the suspension is equipped with bubble levels for all axes, and the cantilever can be adjusted in height.

**Figure 5.**
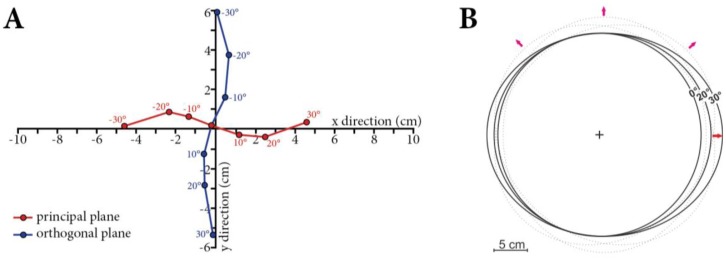
(**A**) Pointing accuracy of the ManTIS. The coordinate system center is aligned to the center of the target; (**B**) Ground instantaneous fields of view (GIFOV) for the range of view zenith angles of the ManTIS. The dotted lines show three view azimuth angles for a constant view zenith angle of 30°. The arrows indicate viewing direction of the foreoptic.

In order to investigate the pointing accuracy of the ManTIS field spectro-goniometer, the sensor sled was equipped with a laser pointer replacing the foreoptic of the spectro-radiometer. Afterwards, a full measurement scheme was carried out and the path left by the laser beam on the surface was recorded. The deviation of the laser beam representing the center of the sensor GIFOV shows values within ±6 cm (Figure 5A). The deviation increases with increasing view zenith angles, indicating that the zenith arc in not perfectly round or that the weight of the sensor sled slightly bends down the freely suspended zenith arc in higher view zenith angle positions.

### 4.3. Ground Instantaneous Field of View and Sensor Self-Shadowing

In its current configuration, the foreoptic of the spectro-radiometer for the target measurements has an IFOV of 8.5°. The distance of the foreoptic to the ground can be slightly adjusted between 1.98 and 2.03 m, and is set currently to 2.02 m. The GIFOV changes with increasing view zenith angle. In the current ManTIS field spectro-goniometer configuration, the maximum view zenith angle is 30°. Therefore, at nadir an almost circular footprint with 30.0 cm occurs that becomes slightly elliptical towards higher view zenith angles reaching a major half axis of 34.8 cm in the 30° view zenith angle position. Figure 5B visualizes the change in footprint area for the main view zenith angle positions of the default measurement scheme. Thus, the spectro-radiometer is always measuring approximately the same surface area in the center of the hemisphere. However, when also including the pointing accuracy of ±6 cm, a homogeneous sampling area of approximately 25 cm in radius around the center of the target plot is needed to acquire representative spectro-goniometer measurements.

Another big issue in spectro-goniometer measurements is abnormalities in the HCRF measurements through sensor self-shadowing which mainly occurs when the foreoptic of the spectro-radiometer is aligned with the sun [14]. This position is also known as the hot spot position. Spectro-directional measurements in this region have to be replaced by simulated data. Hot spots cannot appear in ManTIS spectro-goniometer measurements in the Arctic, since the ManTIS has a maximum view zenith angle of 30° and the illumination zenith angles are always larger than 43° in these geographical positions [40]. Shadowing of the sampling plot by ManTIS parts (zenith arc, suspension) itself is unavoidable, but only minimal through the eccentric position of the zenith arc and the small profile of the obstructing aluminum tubes. Again, the high illumination zenith angles in the Arctic reduce the shadowing of the sampling plot, because the freely suspended zenith arc is mounted high over the ground.

### 4.4. Temporal Illumination Changes and Environmental Influences

Field measurements have a disadvantage compared to laboratory measurements, since in the laboratory the influencing factors on the reflectance anisotropy of a surface can be controlled and narrowed to the canopy geometry, multiple scattering effects and sensor-illumination geometry. In the field, additional environmental factors can affect the measurements which cannot be measured or validated in detail at all times. The main factors are upcoming wind during the measurements, changes in the moisture and temperature regime during the day, plant stress, heliotropic leaf movements, and presence of dew on the canopy in the morning [22,32,41]. Here, only carefulness in the choice of the sampling plot can reduce these measurement errors.

The 61 target measurements of the default measurement scheme should be ideally performed simultaneously, but this is not possible. To reduce short-term temporal changes in irradiation and illumination zenith angle changes between the beginning and end of a measurement scheme, we developed a cos-conical dual-beam approach where two spectro-radiometers simultaneously measure the radiance reflected from the target and the total sky irradiance. Instead of using the recorded irradiance directly, we used the irradiance spectra with the aim to interpolate the radiance measurement of the reference panel to the time of the target measurement. This can be only done under the assumption that changes in the irradiance over the time period affect the radiance measurements of the reference panel to a similar degree in the certain wavelength region [15]. This approach has already been used by various groups, but mostly with a sunphotometer with limited spectral bands for irradiance recording [15,17]. A more detailed consideration of this approach is given in Section 5.1. Moreover, we developed an outlier indicator system showing in which sensor positions higher illumination changes occurred, and therefore stronger interpolation of the reference panel measurement was needed. This shows at which sensor positions more caution is needed in the interpretation of the spectro-directional results.

Another technical challenge is the sun azimuth angle change over time during the beginning and end of the measurements. The measurement scheme is optimized towards the solar principal plane, and a change in the sun azimuth angle leads to a shift in the results. Therefore, the developed software application calculates for each azimuth circle (overall five) within the measurement scheme a correction factor which can be set in the AAM at the chosen view zenith angle position (0°, 5°, 10°, 20°, 30°) before the zenith arc is rotated. This regular manual correction of the solar principal plane during the measurements decreases the divergence between the projected and real principal plane to 0.5°. However, since the geographic north is determined by a compass and manually set in the AAM, the uncertainty between the projected and real principal plane increases to 1.5° to 2°.

## 5. Data Analysis

### 5.1. Data Processing

First, all acquired measurements are transferred into a database and pre-processed. Since the GER-1500 spectro-radiometers produce DN (digital number) values as output, the first pre-processing step is their conversion to radiance and irradiance values with the help of the sensor calibration files provided by the manufacturer as well as the storage in a standard ASCII format. Then automatic quality tests for detecting outliers in the measurement scheme and sensor noise are realized.

In order to derive the HCRF for each viewing positions, Equation (2) has to be adapted. Due to practical reasons, Spectralon® reference panel measurements are performed only from the nadir view direction. Moreover, the used reference panel has an 8° hemispherical spectral reflectance calibration, and therefore a correction factor R_ref_ which ideally corresponds to the BRF of the reference panel. Since it is known that the BRF of the reference panel also depends on the sun zenith angle (θ_i_), a correction factor c_ref_ is replacing R_ref_. This correction factor c_ref_ uses the correction algorithm developed by Sandmeier *et al.* [38] under the assumption that the reflectance anisotropy is nearly invariant between different Spectralon® panels [39]. Equation (3) shows the modified HCRF formula:

(3) $$\text{HCRF}(\lambda ;\theta_{i},\phi_{i};\theta_{r},\phi_{r})=\frac{L_{r}(\lambda ;\theta_{i},\phi_{i};\theta_{r},\phi_{r})}{L_{\text{ref}}(\lambda ;\theta_{i},\phi_{i})}\cdot c_{\text{ref}}(\lambda ,\theta_{i})$$

As mentioned in Section 4.4, the spectro-goniometer measurements at various sensor positions cannot be performed at the same time. Therefore, short-term temporal changes in irradiation as well as illumination zenith angle changes between the beginning and end of a measurements scheme occur. We try to account for these effects by interpolating the reference panel measurement L_ref_ taken at time t_0_ towards the timestamp t_x_ of the actual target measurement L_r_ with help of a weight factor c_diff_. The weight factor c_diff_ is obtained using the diffuse total irradiance measurements of the second spectro-radiometer at the timestamp t_0_ and t_x_ (Equation (4)). Therefore, we assume that changes in the irradiance over the time period affect the radiance measurements of the reference panel to a similar degree in the certain wavelength region [15]:

(4) $$c_{\text{diff}}(\lambda ;\theta_{i},\phi_{i};t_{x})=\frac{E_{\text{diff}}(\lambda ;\theta_{i},\phi_{i};t_{x})}{E_{\text{diff}}(\lambda ;\theta_{i},\phi_{i};t_{0})}$$

In order to evaluate the quality of this approach, we introduced an outlier indicator which uses the continuous irradiance readings and the L_ref_ measurements at the beginning and end of a measurement scheme. The visualization of this outlier indicator helps to interpret the HCRF measurements of the full hemisphere. Thus, the HCRF calculation from ManTIS field measurements results in the following formula (Equation (5)) introducing the relative time span between the L_ref_ measurement in the nadir view position and the L_r_ measurement in the actual viewing position of the measurement scheme:

(5) $$\text{HCRF}(\lambda ;\theta_{i},\phi_{i};\theta_{r},\phi_{r})=\frac{L_{r}(\lambda ;\theta_{i},\phi_{i};\theta_{r},\phi_{r};t_{x})}{L_{\text{ref}}(\lambda ;\theta_{i},\phi_{i};t_{0})\cdot c_{\text{diff}}(\lambda ;\theta_{i},\phi_{i};t_{x})}\cdot c_{\text{ref}}(\lambda ,\theta_{i},t_{0})$$

Only under the assumption that the HCRF is constant over the IFOV of the sensor and that the measurements are taken under clear sky with predominantly direct radiation, the measured HCRF values approximate the BRF. In order to separate the BRDF effects from the underlying surface reflectance characteristics, the HCRF data of one hemisphere has to be normalized by the nadir viewing reflectance signature of the target surface [14,27]. This normalization creates the anisotropy factor (ANIF) Equation (6) [14]:

(6) $$\text{ANIF}(\lambda ;\theta_{i},\phi_{i};\theta_{r},\phi_{r})=\frac{\text{HCRF}(\lambda ;\theta_{i},\phi_{i};\theta_{r},\phi_{r})}{{\text{HCRF}}_{\text{nadir}}(\lambda ;\theta_{i},\phi_{i};\theta_{r}=0,\phi_{r}=0)}$$

With help of the ANIF the spectral-directional characteristics can be compared between different target surfaces or at changing illumination geometry. For an overall estimation of the reflectance anisotropy in a certain wavelength and in order to further analyze the spectral variability of the reflectance anisotropy, Sandmeier *et al.* [14] developed the anisotropy index (ANIX) Equation (7):

(7) $$\text{ANIX}(\lambda ,\theta_{i})=\frac{{\text{HCRF}}_{\max}(\lambda ,\theta_{i})}{{\text{HCRF}}_{\min}(\lambda ,\theta_{i})}$$

The ANIX is the ratio of the maximum HCRF and minimum HCRF of a measured hemisphere, and is calculated for a certain wavelength region as well as a defined azimuth plane [14]. Since reflectance anisotropy is more strongly pronounced in the solar principal plane [14,22] and for easier comparability, the ANIX is usually presented with respect to the solar principal plane.

### 5.2. Data Visualization

**Figure 6.**
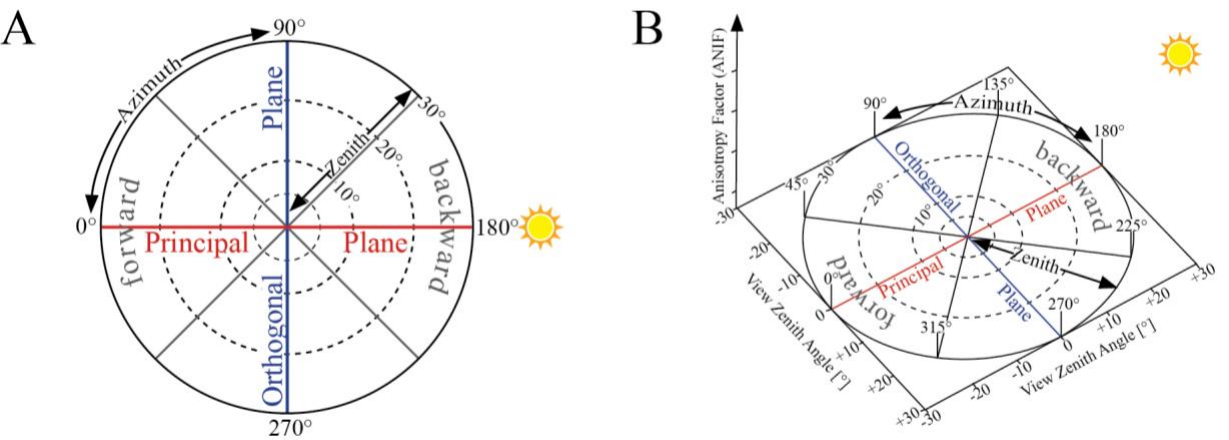
(**A**) Polar coordinate system used for presenting BRDF data in 2D plots; (**B**) Polar coordinate system used for presenting BRDF data in 3D plots.

The processed HCRF data together with metadata of the surface measured at a certain illumination geometry are stored in a database following the recommendations of Sandmeier [22]. For a better interpretation and comparison of the spectro-directional data, the visualization as surface models in 2D and 3D plots is commonly used. Therefore, we aligned a visualization model of Küster [42] coded in the programming language Python to our measurement scheme. To avoid misinterpretation of the visualization, Figure 6 shows the polar coordinate system used for presenting the results.

## 6. Performance of ManTIS Field Spectro-Goniometer in the Field

### 6.1. Test Site and Experiment Setup

**Figure 7.**
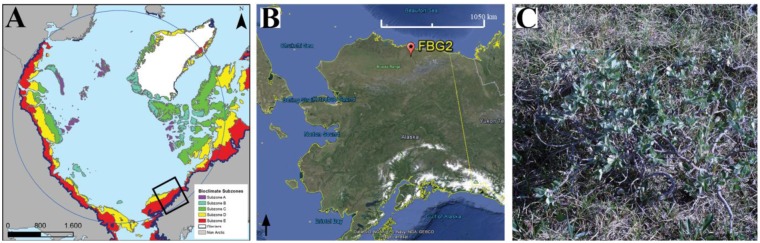
(**A**) The study location in respect to the bioclimate subzones of the circumpolar arctic vegetation map (CAVM) [43]; (**B**) Location of the sample plot FBG2 in the Alaskan Low Arctic. Image Source: Google Earth, 2013; (**C**) Photo of the prostrate dwarf deciduous shrub community measured at solar noon (sun zenith angle of 47°).

The ManTIS field spectro-goniometer was already field-tested on two Arctic expeditions [34,35]. In order to show the quality of field HCRF retrieval and the BRDF analyzing approach, we present a spectro-directional characterization of a low growing vegetation community in a challenging geographic location for field spectro-goniometer measurements. The HCRF data sets of the sample plot at the Franklin Bluffs study location, AK, USA (69°40′28″N, 148°43′15″W, 122 m ASL) were measured in the summer season of 2012 (Figure 7A,B).

The sample plot FBG2 is located in the bioclimate subzone D of the circumpolar arctic vegetation map (CAVM) [43] and is part of the North American Arctic transect (NAAT) established by Walker *et al.* [44]. The dominant vegetation can be described as moist non-acidic tundra (MNT) [45]. The sample plot shows a homogenous coverage with a prostrate dwarf deciduous shrub (*Salix arctica*) community as well as sedges and forbs (Figure 7C). Important to mention is that there are dense and thick moss and lichen mats in the understory, therefore no open soil is exposed. The average vegetation height of the shrub layer is 35 cm, of the sedge and forb layer 15 cm, and of the moss and lichen layer 2 cm. At the day of the measurements (9 July 2012) the vegetation was nearly at the peak of the phenological stadium. Since the sample plot FBG2 is located next to the well-established and researched Franklin Bluffs moist/zonal study site (FB_m/z), a more detailed vegetation description of the study location can be found in Kade *et al.* [46]. Moreover, Buchhorn *et al.* [47] presents a detailed hyperspectral characterization of the Franklin Bluffs study location and MNT vegetation.

**Figure 8.**
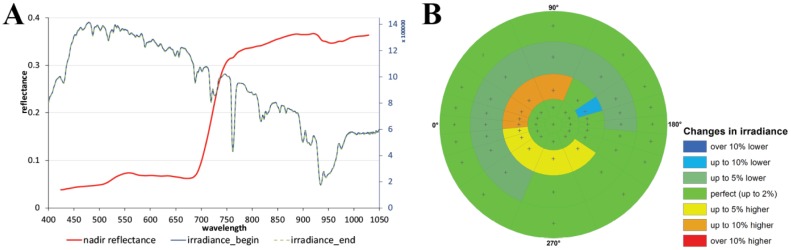
(**A**) Nadir reflectance spectrum and irradiance profiles of the prostrate dwarf shrub-nontussock sedge-moss tundra sample plot FBG2 at the beginning and end of the measurement scheme; (**B**) Polar plot of the outlier indicator showing short-term illumination changes during the measurement scheme.

For the presented case study, a complete hemispherical cycle was measured on a clear-sky day at solar noon (measurements started at 13:48 local time) and under gentle wind conditions. Therefore, the illumination direction had a sun zenith angle of 47° and a sun azimuth angle of 180°. The time needed to complete the measurement scheme with 61 sensor positions was 25 min and therefore not optimal. The sun zenith angle changes between the beginning and the end of the measurement scheme amount to 0.4° and the sun azimuth changes amount to 8°. The HCRF values were calculated following Equation (5). Figure 8A shows the reflectance spectrum as well as the irradiance spectra of the nadir viewing position to the beginning and end of the measurement scheme, where Figure 8B presents the quality assessment of the interpolation approach used to reduce temporal errors. It is notable that in the third measurement circle (10° viewing zenith angle position) of the measurement scheme stronger atmospheric changes occurred which had to be corrected. This region of the ManTIS hemisphere needs more caution in the interpretation of the spectro-directional characteristics of the surface.

### 6.2. Results and Discussion

**Figure 9.**
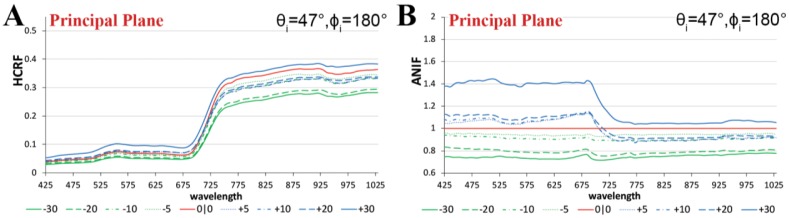
(**A**) HCRF values of the prostrate dwarf-shrub community for various view zenith angles in the solar principal plane; (**B**) Anisotropy factors (ANIF) of the prostrate dwarf-shrub community for various view zenith angles in the solar principal plane.

**Figure 10.**
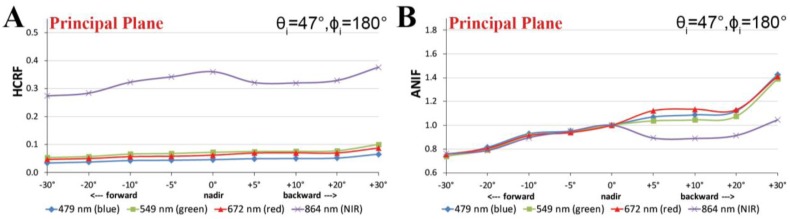
(**A**) HCRF values versus view zenith angles in the solar principal plane. (**B**) Anisotropy factors (ANIF) versus view zenith angles in the solar principal plane.

The spectral HCRF and ANIF data of the prostrate dwarf-shrub community for the main view zenith directions in the solar principal plane are presented in Figure 9. Where in the HCRF data (Figure 9A) changes in the reflectance anisotropy are barely visible, the ANIF data (Figure 9B) exempted from the underlying surface reflectance characteristics show the strong spectral variability in the reflectance anisotropy. The ANIF data show that equal to higher reflectance values compared to the nadir value appear in the backward viewing directions of the solar principal plane, and that the reflectance values in the forward viewing directions are lower. This is especially well observable in Figure 10, where the HCRF and ANIF data for specific wavelengths are presented over the view zenith angles in the solar principal plane. The ANIF values in the solar principal plane range from 1.0 to 1.45 in the visible and 0.9–1.1 in the near-infrared wavelength region of the backward viewing directions, whereas in the forward viewing directions the ANIF values range from 0.7 to 0.95 in the visible to near-infrared wavelength region (Figure 9B). Therefore, a higher degree in reflectance anisotropy occurs in the visible (400–700 nm) than in the near-infrared (700–1,400 nm) wavelength region.

These findings have been also found in other spectro-directional studies of planophile and erectophile vegetation [27,28,31]. The reason for the specific reflectance shape along the view zenith direction is the canopy geometry influencing the distribution and proportion of shadowed and illuminated surfaces within the plant canopy which change under varying viewing-illumination geometries [27,48]. Multiple scattering effects in the vegetation canopy regulate the intensity (“darkness”) of the shadows [27], and create therefore the spectral dependence of the BRDF effects.

Since in wavelength regions with high absorption (visible blue and red chlorophyll absorption bands) the relatively low amount of radiation in the vegetation canopy reduces the multiple scattering effects, the contrast between shadowed and illuminated surfaces increases and therefore enhances the reflectance anisotropy [14]. Vice versa, higher multiple scattering effects in wavelength regions with higher reflection (visible green and near-infrared bands) reduce the contrast in the canopy.

**Figure 11.**
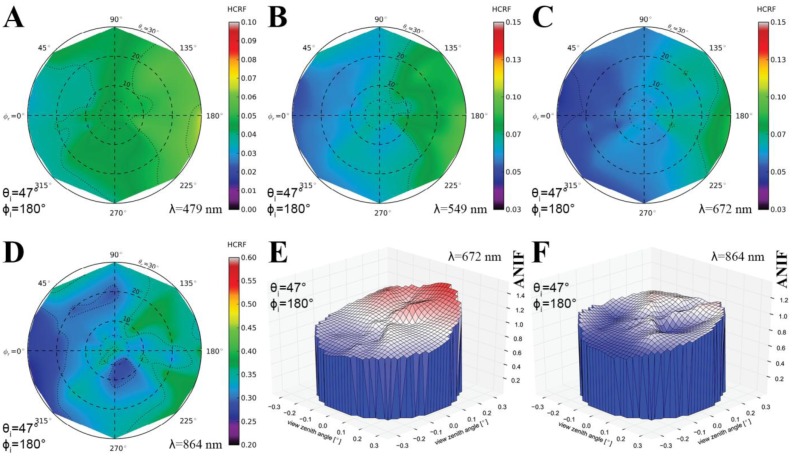
(**A**–**D**) Polar plots of the HCRF data for all view angles at wavelengths of (A) 479 nm; (B) 549 nm; (C) 672 nm; and (D) 864 nm. (**E**–**F**) 3D visualization of the ANIF data for all view angles at wavelengths of (E) 672 nm and (F) 864 nm.

Figure 11A–D shows the visualization of the HCRF data in 2D polar plots for four important wavelengths in the visible blue, green, and red as well as near-infrared spectrum. It is viewable that the BRDF effects in the prostrate dwarf-shrub community are strongest in the solar plane and decrease towards the solar orthogonal plane. Moreover, the distribution of the HCRF values over the hemisphere shows in some viewing positions outliers, especially in the near-infrared wavelength region (Figure 11D). An explanation could be that the vegetation cover of the sampling plot is not perfectly homogeneous due to the chosen prostrate dwarf-shrub community or that the periodic presence of wind has influenced the vegetation canopy and thus the HCRF measurements.

**Figure 12.**
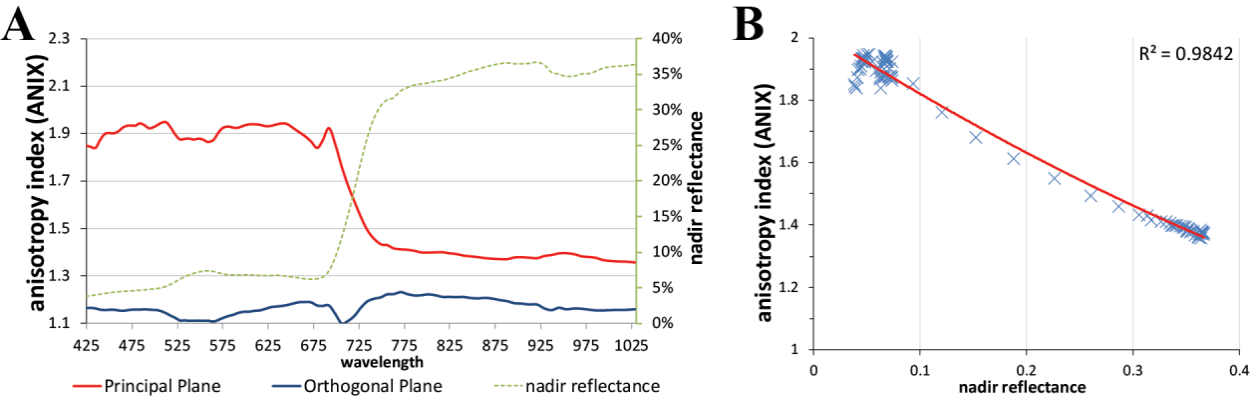
(**A**) Anisotropy index (ANIX) versus wavelength in the solar principal and orthogonal plane; (**B**) ANIX versus nadir reflectance showing strong linkage (higher degree of reflectance in nadir view position = lower degree of reflectance anisotropy).

Figure 11E,F shows the visualization of the ANIF data in 3D of the visible red and near-infrared wavelength spectrum. The differences in the degree of reflectance anisotropy between the visible and near-infrared wavelength region are well notable. A more quantitative analysis of the spectral variability of the BRDF effects of the prostrate dwarf-shrub community allows the plotting of the ANIX over the spectral range for the solar principal and orthogonal plane (Figure 12A). It shows that BRDF effects are pronounced in the solar principal plane and low in the solar orthogonal plane. It also shows an unexpected fact; normally the ANIX graph in the solar principal plane shows a strong dip in the visible green (500–550 nm) because of the increase in multiple scattering though more available radiation in the vegetation canopy, and therefore reduced reflectance anisotropy. However in this case this was not observed. A reason could be that since MNT vegetation does not have a distinct green reflectance peak [47], less multiple scattering appears in this wavelength region. Figure 12B shows the expected linkage of the degree of reflectance anisotropy and degree of reflectance by plotting ANIX against the nadir reflectances of the prostrate dwarf-shrub community.

**Figure 13.**
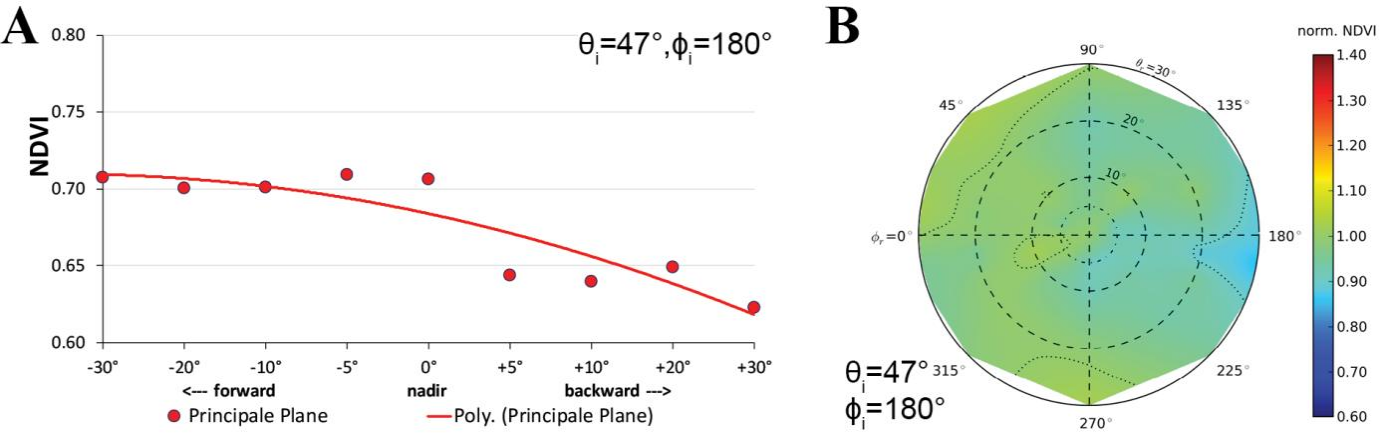
(**A**) NDVI for various view zenith angles in the solar principal plane; (**B**) Polar plot of the nadir normalized NDVI data for all view angles of the dwarf-shrub community.

The calculations of vegetation indices (VI) from spectro-directional data create new functions (called vegetation index distribution functions) [42]. Therefore, the spectral variability of the reflectance anisotropy has impacts on VIs such as the normalized difference vegetation index (NDVI). Especially the NDVI calculated from two wavelength regions (visible red and near-infrared) with completely different BRDF characteristics is affected. Several studies have already researched the influence of BRDF effects on the NDVI in the broadband and hyperspectral domain [29,41,42,49]. In the analyzed prostrate dwarf-shrub community, the NDVI values observed under viewing zenith angle up to ±30° increase towards the forward viewing directions and decrease towards the backward viewing directions in the solar principal plane (Figure 13A). The highest difference is notable in the +30° backward viewing direction of the solar principal plane where the off-view NDVI is 12% lower than in the nadir viewing position. Figure 13B shows the NDVI values of all possible viewing positions within the ManTIS hemisphere normalized to the nadir NDVI.

## 7. Conclusions and Outlook

The availability of ground-based multi-angular RS data is important for the calibration of off-nadir reflection data as well as the potential derivation of canopy structure parameters from remote sensing data. The ManTIS field spectro-goniometer was developed for this purpose, and represents a tool for ground-based multi-angular observations of low-growing vegetated surfaces (up to 1 m vegetation height) which can be used in geographical challenging environments such as the Arctic were heavy or fully automated field goniometers reach their limits.

In this paper, the development of a manual transportable instrument platform for ground-based spectro-directional observations (called ManTIS) and the resultant hyperspectral field spectro-goniometer system has been described. The ManTIS can be equipped with various sensor systems and represent a lightweight, stable, and low-cost platform for spectro-directional observations with up to 30° viewing zenith angle and 360° viewing azimuth angle. The innovative mounting of the zenith arc enables instrument setup on small assembly space. But nevertheless it offers a 2 m distance between the surface target and the sensor in unison with a high angular accuracy and fast execution of the measurements. The platform is equipped in its current configuration as AWI ManTIS field spectro-goniometer with two GER-1500 spectro-radiometers, a GPS receiver, and a video camera system.

This article has presented the sensor configuration, measurement strategy as well as the developed software application for the semi-automatic control of the ManTIS field spectro-goniometer. The current measurement scheme with 61 viewing points was optimized with respect to the solar principal plane and allows the hemispherical conical reflectance factor (HCRF) recording of a ManTIS hemisphere within 18 min under optimal conditions. The pointing accuracy of the system is within ±6 cm and the current instantaneous field of view (IFOV) of the sensor is 8.5°. Therefore, a homogeneous sampling area of approx. 25 cm in radius around the center of the target plot is needed to acquire representative spectro-goniometer measurements. The developed data processing chain in connection with the used software for the semi-automatic control provides a reliable method to reduce temporal effects during the measurements.

The AWI ManTIS field spectro-goniometer was intensely field-tested on two Arctic expeditions and proved its value to characterize the spectro-directional effects of vegetation surfaces. Moreover, this article presented the results of a spectro-goniometer measured Arctic vegetation surface in order to show the high quality and the visualization approaches of the received data. The results of the two expeditions form the start of the systematic spectro-directional characterization of Arctic vegetation communities in order to create a spectral BRDF library, which will be made available to the scientific community. For future measurements it is planned that the field spectro-goniometer system will be improved by a 3D camera system delivering geometric properties of the observed vegetation.

The ManTIS was nationally registered for a patent on 25 October 2011, and internationally registered for a patent on 27 June 2012. The patent publication number is DE 10 2011 117 713.A1 (international publication number: WO2013013652.A1) and it has been published on 31 January 2013 [50,51]. The patent is still pending to the time of the publication of this article. More information is available under http://patentscope.wipo.int/search/en/WO2013013652. Moreover, the ManTIS instrument platform will be produced under license and sold by W. Ludolph GmbH & Co. KG in Bremerhaven, Germany (http://www.ludolph.de/) for the international market.
